# Superior Sinus Venosus Atrial Septal Defect with Partial Anomalous Pulmonary Venous Drainage—Minimally Invasive Approach—Case Report

**DOI:** 10.3390/medicina57090984

**Published:** 2021-09-18

**Authors:** Horațiu Moldovan, Andra-Mădălina Sibișan, Robert Țigănașu, Bogdan-Ștefan Popescu, Gabriel Vasile, Daniela Gheorghiță, Ondin Zaharia, Victor Sebastian Costache, Andrada Guță, Adrian Molnar

**Affiliations:** 1 Faculty of Medicine, Carol Davila University of Medicine and Pharmacy, 014461 Bucharest, Romania; h_moldovan@hotmail.com (H.M.); gabrielvasile882@yahoo.com (G.V.); ondin.zaharia@gmail.com (O.Z.); 2 Clinical Emergency Hospital Bucharest, 014461 Bucharest, Romania; andrasibisan@yahoo.com (A.-M.S.); tiganasu.robert@yahoo.com (R.Ț.); bogdan-stefan.popescu@outlook.com (B.-Ș.P.); 3 Faculty of Materials Science and Engineering, Politehnica University of Bucharest, 060042 Bucharest, Romania; 4 Prof. Dr. Theodor Burghele Clinical Hospital, 050659 Bucharest, Romania; 5 Faculty of Medicine, Titu Maiorescu University, 031593 Bucharest, Romania; victorscostache@gmail.com; 6 “Sf. Constantin” Hospital Brasov, 500388 Brașov, Romania; 7 Sanador Clinical Hospital, 010991 Bucharest, Romania; andrada_guta@yahoo.com; 8 Iuliu Hateganu University of Medicine and Pharmacy, 400012 Cluj Napoca, Romania; adrianmolnar097@gmail.com; 9 Heart Institute, 400001 Cluj Napoca, Romania

**Keywords:** atrial septal defect, minimally invasive repair, atrial reconstruction

## Abstract

The atrial septal defect is, after bicuspid aortic valve disease, the most common congenital cardiac disease present in the adult population. The most common atrial septal defects are the ostium secundum type (75–80%), followed by the ostium primum type (15%). The sinus venosus atrial septal defects (SV-ASD), defined as a communication in the posterior part of the interatrial septum, account for about 5 to 10% of atrial septal defects. Approximately 90% of SV-ASDs are associated with partial anomalous pulmonary venous drainage (PAPVD). The minimally invasive approach has gained ground in the treatment of ASDs, especially those of the ostium secundum type. The sinus venosus type is a relatively uncommon form of ASD, which, when associated with a PAPVD, is considered a complex cardiac malformation, and is usually treated in a classical manner, through median sternotomy. We describe the case of a 45-year-old woman diagnosed in adolescence with SV-ASD with PAPVD, who successfully underwent minimally invasive repair with fresh autologous pericardial patch reconstruction through an anterolateral mini-thoracotomy incision. The patient presented with shortness of breath and fatigue after heavy exertions, episodes of paroxysmal nocturnal dyspnea, palpitations during effort and at rest, and had a history of syncope dating from 17 years previously. Echocardiography revealed an SV-ASD with PAPVD in the right atrium and the intraoperative examination discovered that both right pulmonary veins were draining into the superior vena cava.

## 1. Introduction

The atrial septal defect is, after bicuspid aortic valve disease, the most common congenital cardiac disease present in adult populations. The most common atrial septal defects are the ostium secundum type (75–80%), followed by ostium primum (15%). The sinus venosus atrial septal defects (SV-ASD), defined as a communication in the posterior part of the interatrial septum, account for about 5 to 10% of atrial septal defects [1,2,3,4]. Approximately 90% of SV-ASDs are associated with partial anomalous pulmonary venous drainage (PAPVD) and are traditionally repaired through a full sternotomy [5]. The typical superior SV-ASD variant occurs in the upper posterior atrial septum (rostral and posterior to the fossa ovalis) and results from the communication between the superior vena cava (SVC)–right atrial junction and the left atrium, being almost always associated with anomalous pulmonary venous drainage (usually from the right upper and middle lung lobes into the SVC). Due to the position of the defect, sinoatrial node abnormalities are frequently associated [6,7,8,9,10].

Inferior SV-ASDs occur less commonly, in the postero-inferior portion of the septum, resulting, in this case, from anomalous communication of the right atrium and inferior vena cava with the left atrium. Atrial septal defects affect females more often than males. The diagnosis is usually made with echocardiography, this being the preferred method [11,12,13].

ASDs were traditionally treated through median sternotomy, with well-known disadvantages. The minimal approach reduces blood loss, postoperative mechanical ventilation time, duration of intensive care stay, and overall hospitalization time. The cosmetical results are also superior [5].

## 2. Case Report

We describe the case of a 45-year-old woman diagnosed in adolescence with a sinus venosus atrial septal defect (SV-ASD) with partial anomalous pulmonary venous drainage (PAPVD). After postponing the surgical treatment due to fear of surgical intervention, the patient presented with shortness of breath and fatigue after heavy exertions, episodes of paroxysmal nocturnal dyspnea, palpitations both during effort and at rest and had a history of syncope dating from 17 years previously. A series of tests were carried out to determine whether the patient was fit to undergo cardiac surgery to close the septal defect. The patient was diagnosed with heart failure NYHA (New York Heart association) functional class III.

During the physical examination, the patient presented with a typical “gracile habitus”, being thin for her height, with normal skin color and a 95% blood oxygen saturation. She had normal blood pressure (119/80 mmHg) and a good volume pulse. The patient also had a loud S1 and an ejection systolic murmur in the Erb’s point. The jugular venous pressure was not particularly elevated and the chest X-ray was clear. Blood tests showed a hemoglobin concentration of 14.4 g/dL and a normal brain natriuretic peptide concentration of 22.4 pg/mL. There was no evidence of decompensated heart failure or peripheral oedema.

The patient’s preoperative electrocardiography (ECG) at rest demonstrated an inferior atrial rhythm, a right QRS axis deviation (+100 degrees), a narrow QRS complex and a minor right bundle-branch block. The abnormal P-wave axis (negative in leads DII, DIII and aVF) indicated that the depolarization of the atrium was not initiated in the sinus node (ectopic atrial rhythm), suggesting an underlying sinus node disease. The chest X-ray showed no evidence of cardiomegaly, which would have been a prognostic marker for a poor chance of survival.

**Figure 1:**
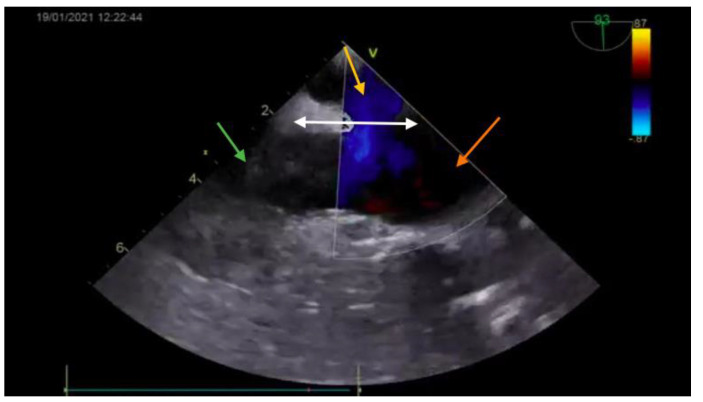
Preoperative echocardiography (bicaval view—Doppler; SVC—green arrow; right atrium—orange arrow; interatrial septum—white arrow) showing the ASD (yellow arrow).

**Figure 2:**
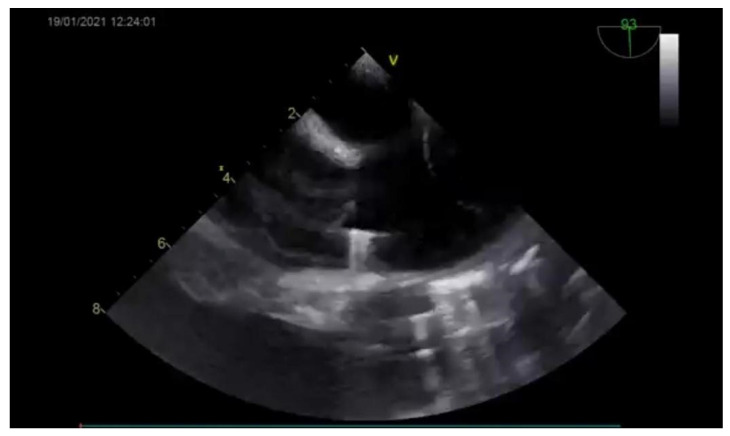
Preoperative echocardiography (bicaval view) showing the ASD.

The diagnosis was based on the transthoracic echocardiography (Figure 1 and Figure 2). It revealed an SV-ASD with PAPVD which appeared to open in the right atrium, a dilated right atrium and ventricle, and a left-to-right shunt with Qp:Qs of 2.3 (Qp-pulmonary flow/Qs-systemic flow). The patient had normal pulmonary artery pressure, which was measured during echocardiography. 

After careful examination, the patient was deemed fit to undergo cardiac surgery. Given the cosmetic concerns of the patient and considering her young age, a minimally invasive approach was preferred.

The incision was made in the fourth right intercostal space. Cardiopulmonary bypass with aortic cross-clamping was used with bicaval and aortic peripheral cannulation. Cardiac arrest was induced with Brett-Schnider cardioplegia at moderate systemic hypothermia (at 32 degrees Celsius).

**Figure 3:**
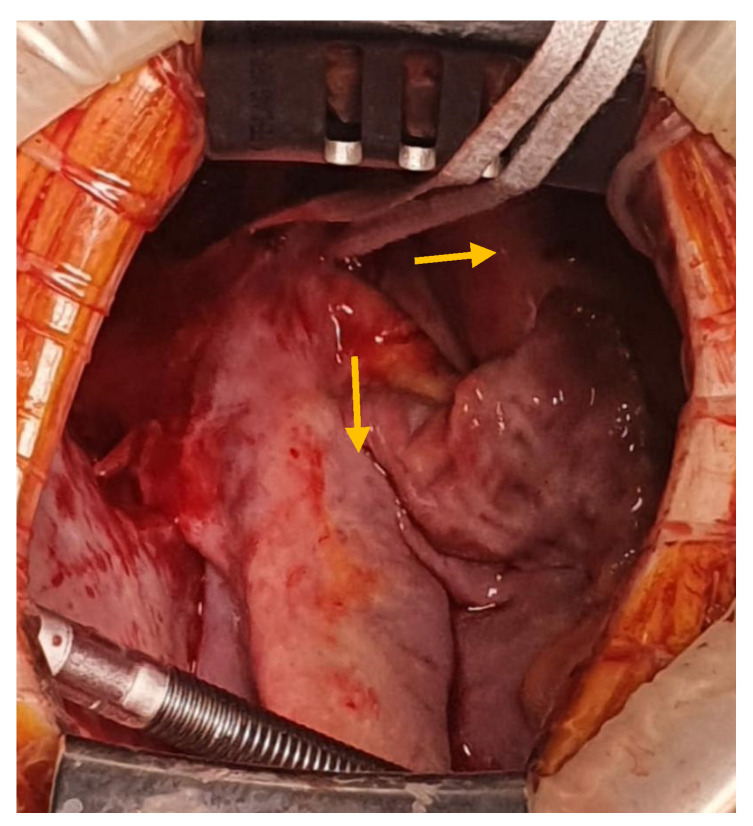
View of PAPVD in the SVC (pulmonary veins in the SVC—yellow arrow).

**Figure 4:**
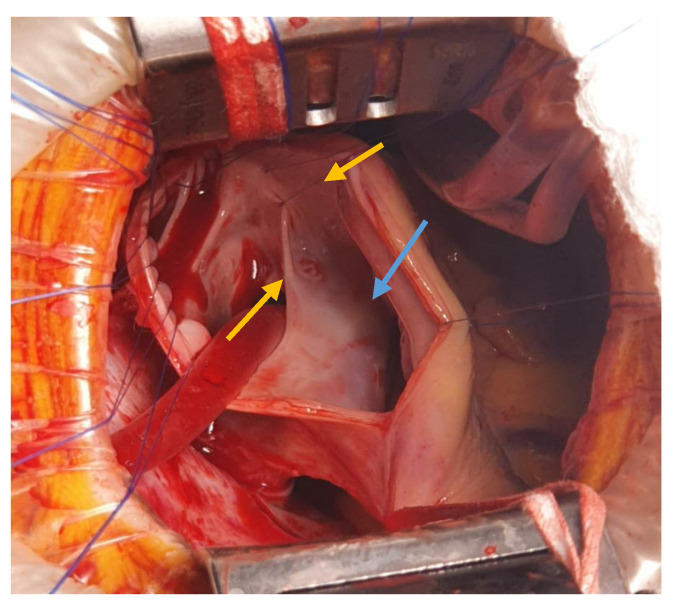
Intra-atrial look of the SV-ASD with PAPVD (pulmonary veins ostia—yellow arrows; ASD—blue arrow).

The interatrial defect was exposed through a right atriotomy extended across the cavoatrial junction into the superior vena cava. The examination of the right atrium cavity revealed an atrial septal defect in the proximity of the SVC and both right pulmonary veins draining into the SVC, proximally (Figure 3 and Figure 4).

**Figure 5:**
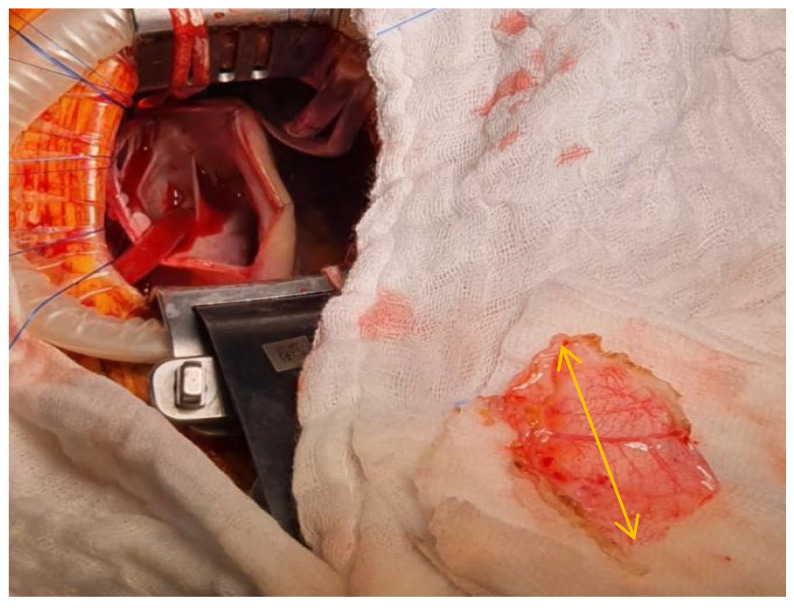
Pericardial patch sizing (pericardial patch—yellow arrow).

A fresh autologous pericardial patch (Figure 5) was sutured in with a fine monofilament suture to close the ASD and to redirect the pulmonary venous flow towards the left atrium.

**Figure 6:**
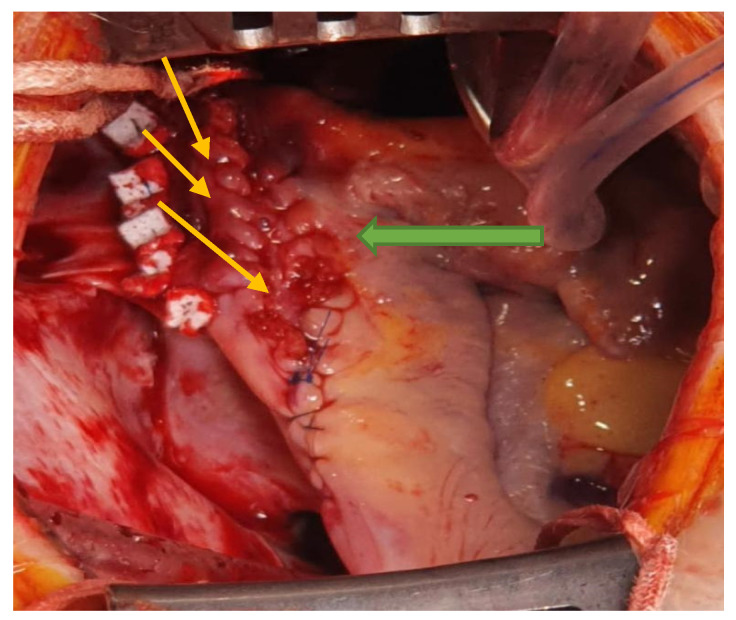
Right atrial and SVC enlargement plasty, reinforced with PTFE patches (yellow arrows), using a fresh autologus pericardial patch (green arrow).

In order to prevent the SVC narrowing, a right atrial and SVC enlargement plasty was performed using a fresh autologous pericardial patch, reinforced with PTFE (Poly-Tetra-Fluoro-Ethylene) patches (Figure 6). 

**Figure 7:**
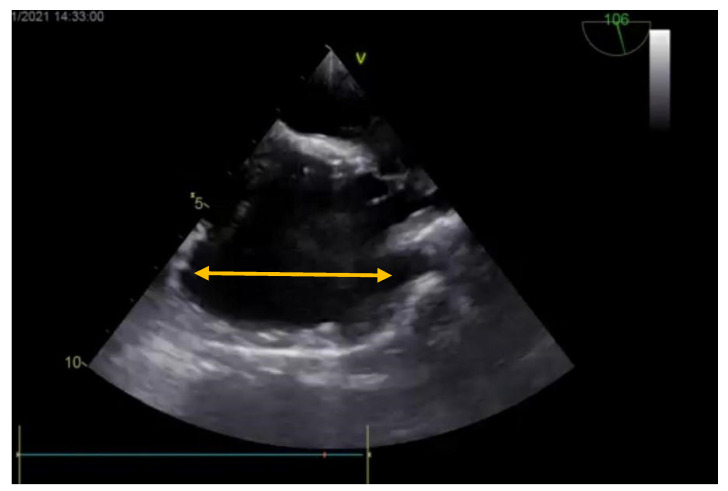
Postoperative echocardiography (bicaval view) showing the closed ASD and the plasty enlarging the SVC and right atrium (yellow arrow).

**Figure 8:**
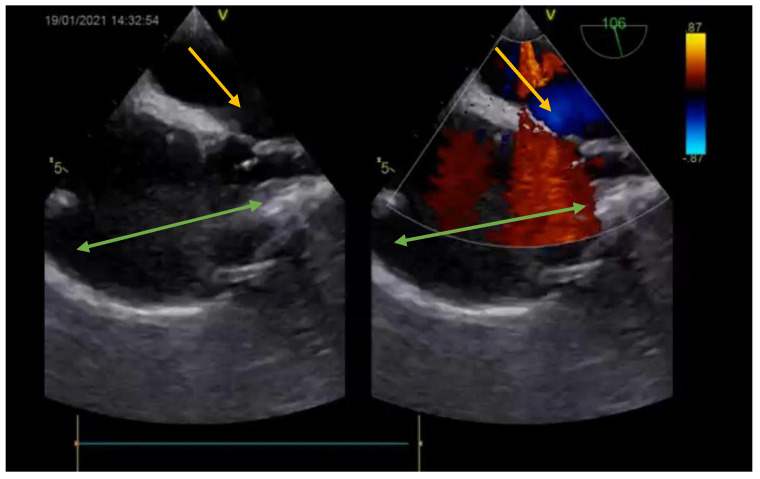
Postoperative echocardiography (bicaval view—Doppler) showing the closed ASD (no leaks—yellow arrow) and the enlarged SVC and right atrium (green arrow).

Postoperative transesophageal echocardiography (Figure 7 and Figure 8) showed the right heart cavities with no dilation and a minimum degree of tricuspid valve functional regurgitation. The inferior vena cava had a 19 mm diameter, and no residual shunts. Also, there was a small accumulation of fluid in the pericardium. The first ECG showed a normal sinus rhythm with 75 bpm, QRS axis at +120 degrees, minor right bundle-branch block with negative T waves in V1, V2. The patient later developed an accelerated junctional rhythm with 50 bpm and narrowed QRS complex, as is characteristic for a minor right bundle-branch block. The pulmonary artery pressure (measured during echocardiography) was normal. 

The patient was extubated 5 h after surgery, with a minimum pneumopericardium, and, after removing the drainage tubes, developed a pneumothorax that required percutaneous insertion of a tube in the pleural space. After the remission of the pneumothorax, the tube was removed. The patient made a good recovery and was discharged on the 7th postoperative day. Medical treatment was recommended with bosentan and aspirin.

## 3. Discussion

Surgical techniques for ASD repair have developed since the beginning of cardiac surgery, in the late 1940s. Regarding the SV-ASDs with PAPVD, the main techniques that have been historically established are the two-patch technique (or baffle technique) and the Warden procedure, depending on the pulmonary drainage position [14]. Each has its own advantages and risks; the Warden technique avoids the risk of sinus node dysfunction and cavoatrial incision, whereas the two-patch technique does not carry the risk of SVC obstruction—a rare but serious possible complication with these types of repair [15]. In our clinic the minimal invasive approach is the preferred method for treating ASDs, as well as mitral and tricuspid valve surgeries. These cases have always evolved properly and there have been no conversions to the classic approach (median sternotomy).

The newest aspect of surgical ASD repair is robotic technology. Avoiding the thoracotomy incisions and the spreading of the ribs, this approach is said to reduce the pain caused by intercostal nerve damage, reduce postoperative recovery time, and to give greater overall patient satisfaction, as well as better cosmetic results, though it certainly has its limitations. First of all, the technique is challenging and time-consuming, with cardio-pulmonary bypass and aortic cross-clamping times that have been noted to be almost double what they are in the port-access approach. Secondly, when discussing robotic surgery, costs are still a significant barrier, and the duration of the hospital stay of patients has not been significantly reduced compared to the mini-thoracotomy approach. In addition to the above, the absence of tactile feedback as a consequence of using robotic assistance could make a simple suture a technically difficult surgical procedure, which might increase the risk of ASD recurrence.

Representing a complex subset of ASDs, SV-ASDs with PAPVD have traditionally been repaired surgically through a median sternotomy incision [16]. A minimally invasive surgical approach via anterolateral right mini-thoracotomy has been associated with less blood loss, a shorter hospitalization period, faster recovery, improved healing, and better cosmetic results [17]. Furthermore, having this type of incision leaves open the possibility for future median sternotomy with fewer adhesions, given the fact that the dissection and tissue trauma are limited [18].

Patients with SV-ASD demonstrate unique anatomical and surgical features, and are at risk from postoperative complications [19]. Postoperative sinus node dysfunction is common in patients after SV-ASD repair, potential mechanisms including an anatomic anomaly of the sinus node, intrinsic sinus node dysfunction, or surgical trauma caused by the proximity of the SV-ASD to the sinus node, the internodal tracts and the blood supply for the sinus node. A very significant complication that has been noted with the port-access approach is damage to the phrenic nerve, which could be avoided by carefully establishing the path of the phrenic nerve and opening the pericardium 2 cm away from it. Topical cooling would also have to be avoided [20].

In the past three decades, mortality from congenital heart disease has decreased. In general this has been due to various improvements in medical and surgical management, and to technological upgrades in extracorporeal circulation [21]. Regarding imaging techniques, it is recommended to perform transesophageal echocardiography (TEE), not only pre-operatively for diagnostic purposes, but also intraoperatively, due to its ability to help establish the operative plan and to assess the hemodynamic parameters during cardiac de-airing [22]. It was believed that, despite the complexity of the lesion, repair of SV-ASD with PAPVD is associated with low morbidity and mortality even in patients older than 40 years [23]. Furthermore, recent studies have concluded that delaying repair until the third decade of life is frequently associated with a decrease in life expectancy [24]. This case report aims to demonstrate that in experienced centers, patients with isolated complex ASD lesions, such as an SV-ASD with PAPVD, can be repaired using a minimally invasive surgical approach.

## 4. Conclusions

To conclude, surgical repair of the SV-ASD involves well-known techniques which have proven to be effective. In the superior type of SV-ASD with PAPVD, which is either in the proximity of the cavoatrial junction or on it, the preferred method of treatment involves creating a pulmonary baffle and making a pericardial patch enlargement of the right atrium and SVC, whereas a distal placement of the pulmonary drainage or an inferior type of SV-ASD would benefit more from the Warden technique. In this case, the SV-ASD was closed and the PAPVD redirected to the left atrium with an SVC and right atrium baffle. Intraoperative transesophageal echocardiography confirmed the desired surgical result. Furthermore, the minimally invasive approach has shown itself to be a valid option, improving cosmetic results, as well as recovery time.

## Data Availability

The study did not report any data.
